# Intraosseous Ganglion Cyst of the Lunate

**DOI:** 10.5812/kowsar.22517464.2705

**Published:** 2012-01-15

**Authors:** Shahram Nazerani, Adel Ebrahimpour, Arvin Najafi, Ehsan Shams Koushki

**Affiliations:** 1 Department of Surgery, Tehran University of Medical Sciences, Tehran, IR Iran; 2 Shahid Beheshti University of Medical Sciences, Tehran, IR Iran; 3 Trauma Research Center, Baqiyatallah University of Medical Sciences, Tehran, IR Iran

**Keywords:** Bone Cysts, Ganglion Cysts, Lunate Bone, Wrist

## Abstract

Intraosseous ganglia can affect the carpal bones of the hand and must be considered in the differential diagnosis of wrist pain. A 38-year-old female presented with a 14-month history of left wrist pain and a radiolucent cystic lesion was seen computed tomography (CT) scanning. Characteristic radiographic findings of a cyst in association with a fine sclerotic rim was apparent. We report an unusual presentation of a ganglion cyst in the lunate bone with excellent treatment outcome.

## 1. Introduction

Intraosseous ganglion cysts (IGC) of the carpal bones located in the lunate are among the rarely seen pathologic conditions.(1) Carpal bone cysts are frequent, particularly if the subchondral cysts presenting in osteoarthritic and rheumatoid affected wrists are included.(2) On the other hand detecting a single radiolucent lesion in the lunate accompanied by pain is rare. Differential diagnosis of symptomatic cystic lesions of the lunate bone include osteoid osteoma, giant cell tumor, enchondroma, intraosseous ganglion, osteoblastoma and Kienbock’s disease. Isolated cases of ganglion cysts occurring in the lunate, scaphoid, pisiform, hamate, triquetrum, capitate, metacarpal, and phalanx have been reported.(3) The is the most common location because about 70% of hand tissue-gangila arise from the posterior side of scapholunate ligament. Etiology remains largely unknown, however trauma, herniation of the joint capsule, mucoid degeneration, intramedullary metaplasia of mesenchymal cells, and congenital rests of synovial producing cells have been suggested to play a role.(4) Ganglion cysts are seen in all ages but are most frequent during the second, third, and fourth decades of life. We describe a patient with an intraosseous ganglion cyst of lunate bone.

## 2. Case Report

**Figure 1.**
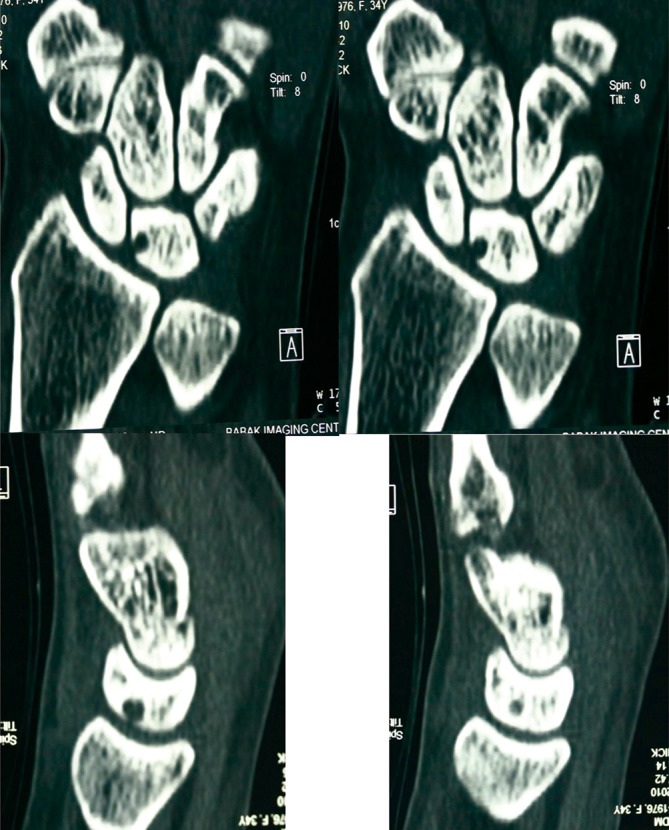
CT scan view of the ganglion cyst

**Figure 2.**
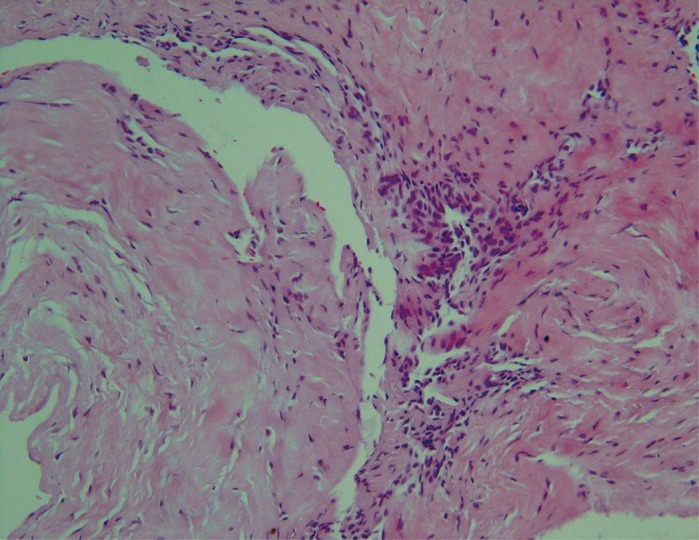
Microscopic view of the ganglion cyst

A 38-year-old female presented with a 14-month history of left wrist pain and a radiolucent cystic lesion found on conventional radiographs and computed tomography (CT) scanning (Figure 1). The patient suffered from an aching pain worsened by usual activity of the left upper extremity and fairly relieved by rest. She denied any prior history of specific trauma. On physical examination except for a vague local tenderness in dorsum of the left wrist she had no sign of synovitis and the range of motion of both wrists was normal. In spite of conservative management such as splinting and local steroid injection and use of NSAIDS she had no improvement. On the radiographs within the lunate beside the scapholunate ligament a radiolucent lesion with a sclerotic margin without collapse of the lunate was detected. CT confirmed a cystic lesion with normal appearance of other parts of the lunate. The patient was operated using a vertical dorsal approach; both the lunate and scaphoid were exposed. Grossly a soft-tissue ganglion anatomically in communication with the lunate intraosseous ganglion was seen through a defect on the posterior side of the lunate. The cyst was evacuated; typical gelatinous ganglion fluid was curetted and the lining membrane was removed. Then the cavity was packed using cancellous bone from the distal of the radius. After closing the joint capsule and wound a removable extension cast was applied for six months. The pathology report described a cystic lesion with a delicate wall of fibro-connective tissue cells without true epithelial lining. Gross and microscopic findings were characteristics of an intraosseous ganglion cyst (Figure 2). The patient was successfully treated surgically After a nine-month follow up period she was asymptomatic without limitation of left wrist motion.

## 3. Discussion

The intraosseous ganglion was described by Fisk(5) in 1949 as a periosteal ganglion-like lesion developing a cystic bony defect through intraosseous penetration. In 1966 Crabbe(6) named it the intraosseous ganglion cyst. Synonymous terms include synovial bone cyst, juxta-articular bone cyst, ganglionic cystic defect of bone and subchondral bone cyst. The differential diagnosis of a lytic painful mass in the lunate consists of chondroblastoma, enchondroma, fibrous dysplasia, osteoblastoma, osteoid osteoma, giant cell tumor, chondromyxoid fibroma, Kienböck’s disease, unicameral bone cyst, osteoarthritis, rheumatoid arthritis and intraosseous ganglion cyst. The first four diagnoses listed differ from intraosseous ganglia by their radiographic presentation of intralesional calcifications; the giant cell tumor is generally poorly demarcated on radiographic examination, whereas intraosseous ganglia show a radiolucent area surrounded by a sclerotic rim.(4) Most intraosseous ganglion cysts are located in the epiphysis of long bones; the predisposition of chondromyxoid fibromas and unicameral bone cysts is the metaphyseal location. In contrast to Kienböck’s disease leading to avascular necrosis of the entire lunate, the intraosseous ganglion cyst only affects a tiny part of the bone. In most cases of rheumatoid and osteoarthritis, signs of degeneration are often seen in painful joints whereas intraosseous ganglion cysts show no signs of degeneration. The most prevalent sites for intraosseous ganglia are the epiphysis of long bones, often present beside a weight-bearing joint such as the knee, hip and ankle. It is more prevalent in females. IGC has a delicate wall of flattened, fibro-connective tissue cells without a true epithelial lining. This type of cyst contains a highly viscous clear mucin consisiting of high concentration of hyaluronic acid in combination with glutamine, albumin and glucosamine. Although there have been several theories about the origin of the IGC, the main etiology is still unclear. There seem to be two fundamental types of intraosseous ganglia: one originating by penetration of an extra-osseous ganglion into the underlying bone, the other being idiopathic.(7) Erosion of an extraosseous ganglion through bone is the generally agreed-on mechanism for the penetrating type.(4) The primary or idiopathic type has no apparent extraosseous communication. It seems that idiopathic type of ganglion cyst originates from modified mesenchymal or synovial cells at the capsule-synovial interface in response to repeated minor injury, explaining high prevalence of ganglion cyst in the scapho-lunate site where the motion and force is concentrated. Repetitive minor trauma and mechanical stress cause intramedullary vascular disturbance and consequently aseptic bone necrosis. Then proliferation of fibroblasts and histocytes and production of hyaluronic acid with mucoid degeneration during tissue revitalization occur to form a cyst. IGC often presents as a painful nodule in the palmar or dorsal aspect of the wrist. Surgery is indicated, if periodical radiography shows significant progression of the cyst or cortical erosion or the patient suffers from continued pain.(8) However, conservative management is beneficial when no cortical erosion or change in size of the cyst is found during follow up . The most important complication of this lesion is pathologic or traumatic fracture. Treatment of IGC is curettage of the cyst and saline solution injection and packing the cavity with cancellous bone graft. In addition to this common approach some alternative surgical techniques such as excision of the lunate, dorsal flap arthroplasty, prosthetic replacement, radiocarpal or intercarpal fusion are also used.(1) In patients with chronic wrist pain along with a cyst with fine sclerotic margins aside the scapholunate ligament the IGC should be suspected. Although CT scanning shows the bony architecture MRI may better demarcate the tissues surrounding the bones. Recurrence has been reported but is rare.(9) We found no recurrence during the 15 month follow-up period.

## 4. Conclusions

Intraosseous ganglion cysts of the carpal bones located in the lunate are one of the rarely seen pathologic conditions. This case had an unusual presentation in the lunate bone with excellent treatment outcome.
